# Valedictory

**Published:** 1876-10

**Authors:** W. F. Westmoreland


My connection with the Atlanta Medical and Surgical Journal, both as editor and proprietor, terminated with the September number. Since its first issue, now more than twenty years, I have been, either as editor or proprietor, more or less actively connected with its publication. I am worn down, and need rest from the harrassing demands incident to the position of editor and proprietor of such a publication.

My leisure in the future will be devoted to literary pursuits in connection with my profession more in unison with my taste, and of which the profession will in due time be informed.

My brother, Dr. J. G. Westmoreland, who with this number takes charge of the Journal as editor, is not a stranger to journalism, as he was for years in the past connected with the Journal, both in its editorial and business management.

I feel confident that the patrons of the Journal will be benefited by the change, and predict for it a brilliant future

W. F. Westmoreland.

